# Comparative analysis of potassium deficiency-responsive transcriptomes in low potassium susceptible and tolerant wheat (*Triticum aestivum* L.)

**DOI:** 10.1038/srep10090

**Published:** 2015-05-18

**Authors:** Li Ruan, Jiabao Zhang, Xiuli Xin, Congzhi Zhang, Donghao Ma, Lin Chen, Bingzi Zhao

**Affiliations:** 1 State Key Laboratory of Soil and Sustainable Agriculture, Institute of Soil Science, Chinese Academy of Sciences, Nanjing 210008, China; 2 Institute of Soil and Water Resources and Environmental Science, College of Environmental & Resource Sciences, Zhejiang University, Hangzhou 310058, P.R. China

## Abstract

Potassium (K^+^) deficiency as a common abiotic stress can inhibit the growth of plants and thus reduce the agricultural yields. Nevertheless, scarcely any development has been promoted in wheat transcriptional changes under K^+^ deficiency. Here we investigated root transcriptional changes in two wheat genotypes, namely, low-K^+^ tolerant “Tongzhou916” and low-K^+^ susceptible “Shiluan02-1”. There were totally 2713 and 2485 probe sets displayed expression changes more than 1.5-fold in Tongzhou916 and Shiluan02-1, respectively. Low-K^+^ responsive genes mainly belonged to the categories as follows: metabolic process, cation binding, transferase activity, ion transporters and so forth. We made a comparison of gene expression differences between the two wheat genotypes. There were 1321 and 1177 up-regulated genes in Tongzhou916 and Shiluan02-1, respectively. This result indicated that more genes took part in acclimating to low-K^+^ stress in Tongzhou916. In addition, there were more genes associated with jasmonic acid, defense response and potassium transporter up-regulated in Tongzhou916. Moreover, totally 19 genes encoding vacuolar H^+^-pyrophosphatase, ethylene-related, auxin response, anatomical structure development and nutrient reservoir were uniquely up-regulated in Tongzhou916. For their important role in root architecture, K^+^ uptake and nutrient storage, unique genes above may make a great contribution to the strong low-K^+^ tolerance in Tongzhou916.

Potassium (K^+^) as the most important macronutrients in the field, takes up about 2 to 10 percent of the total dry weight in plants^1^. As is known to all, K^+^ contributes a lot to enzyme activation, osmoregulation, protein synthesis, electrical neutralization, photosynthesis and control of turgor pressure^2^. Thus K^+^ deficiency brings about many negative impacts on plants, such as: growth inhibition on account of inappropriate osmotic pressure; nutrition imbalance due to the arrest of photosynthesis and protein synthesis; decrease in pathogen resistance and so on.

K^+^ deficiency has a far-reaching influence on agricultural. However, there are large numbers of K-deficient farmlands throughout the world. For example, 75% of the paddy soils in China are K deficient, as well as 67% of the wheat fields in Southern Australia^3^. Around the root zone, the concentration of soil potassium is generally less than 0.3 mM^4^. Thus most plants will face low-K^+^ stress in the process of growth. Most plants can resist low-K^+^ stress, mainly because they have set up strategy to acclimate to low-K^+^ stress. The main reason for plant adaption to low-K^+^ conditions is that they can keep a steady level of K^+^ in the cells and tissues via K^+^ transport^5^. Many high affinity potassium transporter genes, such as *AtHAK5*, *HvHAK1* and *OsHAK1*, were induced by low K^+^ stress^6^,^7^,^8^. Recently, from monocotyledons, OsAKT1^9^ and OsHAK5^10^ have been functionally characterized in plant to demonstrate their key roles in K acquisition from low K supplied culture medium. What’s more, gene microarray analysis of *Arabidopsis* and rice identified that many candidate genes, including jasmonic acid-related enzymes, cell wall proteins, Ca^2+^ signaling proteins, protein kinase and ion transporter families, were induced by low K^+^ stress^6^,^11^,^12^.

Microarray technology, as a convenient tool, allows us to approach gene expression profiles of different plants in various external environments. For example, the transcriptome features in wheat responses to heat^13^, low temperature^14^, drought^15^ have already been detected by microarray technology. The results of microarray have indicated that the wheat transcriptome changes in responses to the above abiotic stress. Thus we can hypothesize that the gene expression levels of wheat may also change in response to K^+^-deficiency. Nevertheless, scarcely any development has been promoted in wheat transcriptional response to K^+^-deficient conditions. Wheat is a main grain crop, whose planting area, total output and total trade volume rank first in all types of crops. Therefore, it’s necessary to make clear the molecular mechanisms of wheat in response to K^+^-deficient conditions.

In the present study, Affymetrix GeneChip of wheat was used to investigating gene expression changes of wheat roots under K^+^-deficient conditions. In order to uncover the key genes in wheat adaptation to low-K^+^ stress, we selected two wheat genotypes which had different low-K^+^ tolerance for transcriptome analysis. “Tongzhou916” is tolerant to low K^+^ stress, whereas “Shiluan02-1” is susceptible to K^+^-deficiency. We investigated the function classification of differentially expressed genes and made a comparison of K^+^-deficiency responses between Tongzhou916 and Shiluan02-1. These results will provide a basis for our better understanding of molecular adaptation mechanisms in different wheat genotypes under K^+^-deficiency.

## Results

### Different sensitivity to K^+^ deficiency

**Figure 1:**
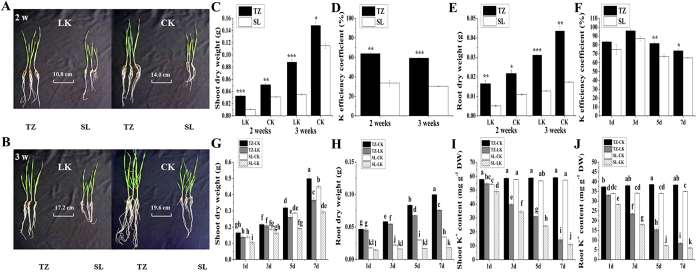
Different sensitivity to K^+^ deficiency between two wheat cvs (K-efficient Tongzhou916 (TZ), K-inefficient Shiluan02-1(SL)). Phenotypes of wheat seedlings during K^+^-sufficient (CK) and K^+^-deficient (LK) treatments for two weeks (**a**) and three weeks (**b**). Shoot biomass (**c**), K efficiency coefficient (**d**) and root biomass (**e**) of the two wheat genotypes under CK and LK conditions for two and three weeks. K efficiency coefficient (**f**), shoot biomass (**g**), root biomass (**h**), shoot K^+^ content (**i**) and root K^+^ content (**j**) of the two wheat genotypes at indicated times (after hydroponically grown in CK conditions for three weeks, wheat seedlings were transferred to CK and LK solutions for 1d, 3d, 5d and 7d, respectively). Data are means ± SE (n = 3). For (**c**), (**d**), (**e**) and (**f**) *, ** and ***denote significant differences at *P* < 0.05, 0.01 and 0.001, respectively. For (**g**), (**h**), (**i**) and (**j**), different letters denote significant differences at *P* < 0.05.

To investigate different genotype responses to K^+^ deficiency in wheat roots, we used two wheat cvs (K-effcient Tongzhou916, K-ineffcient Shiluan02-1) which had marked differences in sensitivity to K^+^ deficiency and root morphology. There were considerable phenotypic differences between Tongzhou916 and Shiluan02-1 during K^+^-sufficiency and K^+^-deficiency (CK and LK) for both two and three weeks (Fig. a,b). Though K^+^ deficiency inhibited growth of the two genotypes, Tongzhou916 had better shoot and root biomass under LK conditions (Fig. c,e), especially in three weeks. K efficiency coefficient was used to evaluate the K^+^-deficiency tolerance of the two wheat genotypes. The K efficiency coefficient of Tongzhou916 were considerably higher than that of Shiluan02-1 (Fig. 1d), indicating that Tongzhou916 had better tolerance to K^+^ deficiency. The differences in K efficiency coefficient between Tongzhou916 and Shiluan02-1 were more marked in three weeks. To make sure that the wheat seedlings were more sensitive to the changes in external K^+^ levels and the differences between the two wheat genotypes were more marked, we chose 3-week-old wheat seedlings, which required more potassium absorbed from the external environment and exhibited more considerable distinctions between the studied wheat genotypes.

To study root transcriptional responses to K^+^ deficiency in Tongzhou916 and Shiluan02-1, firstly we need to ascertain the suitable period applied for the K^+^-deficient treatment. The K efficiency coefficient of Tongzhou916 was higher than that of Shiluan02-1 (Fig. 1f) at all times, indicating that Tongzhou916 had better tolerance to K^+^ deficiency. K efficiency coefficient began to decrease at 3 d. More remarkable differences between Tongzhou916 and Shiluan02-1 appeared at 5d. Moreover, the K^+^ content and biomass were both significantly decreased at 5d time point (Fig. 1g–j). Thus we used five days as the period of K^+^-deficient treatment to analyze variations in wheat root transcriptome during K^+^-deficient conditions.

### Identification of differentially expressed genes in the two genotypes

The Wheat Genome Array used in this study is Affymetrix GeneChip® (Affymetrix, USA). In the present research, we selected two wheat genotypes to analyze the transcriptom changes during K^+^-deficient conditions. Since root is the first organ of plant which can detect element deficiency from the external environment, we harvested the root samples from K^+^-deficient and K^+^-sufficient treatments at 5d. We extracted total RNA of the harvested roots to carry out the following microarray experiments. In order to reduce the errors caused by the biological differences, we selected twenty wheat seedlings with uniform growth and collected roots of these seedlings to combine into one sample. In order to make sure that the microarray data is reproducible and reliable, we used three biological replications (each biological replication contained twenty individuals) for each experimental treatment. We detected over 61200 of the probe signals from Wheat Genome Array. To include more K^+^-deficiency responsive genes as possible, we used 1.5-fold (*P* < 0.05) for further analysis. To assess the reproducibility of microarray data, the correlation coefficients of biological replicates were calculated using GeneSpring GX 11 software. All correlation coefficients of each biological replications were greater than 0.94 (Supplementary Fig. S3A). From the cluster analysis, the microarrays of TZ-LK, TZ-CK, SL-LK and SL-CK clustered into one group, respectively (Supplementary Fig. S3B).

There were 2713 and 2485 differentially expressed genes (DEGs) in Tongzhou916 and Shiluan02-1, respectively (Table 1). Among these 2713 genes in Tongzhou916, there were 1321 up-regulated genes and 1392 down-regulated genes, up to 48.69% and 51.31% of the total DEGs, respectively. Among these 2485 genes in Shiluan02-1, 1177 genes were up regulated and 1308 genes were down regulated, accounting for 47.36% and 52.64% of the total DEGs, respectively. In both wheat genotypes, the number of down-regulated genes was in a slight advantage than that of up-regulated genes. This result was quite similar to the previous study, indicating that the expression levels of many genes in wheat might be inhibited to slow down the speed of wheat growth, whereas other genes were stimulated in order to acclimate to K^+^-deficient conditions^12^. In this study, there may be more up-regulated genes in Tongzhou916 than that in Shiluan02-1 at 5d, which may have an effect on the higher K^+^-deficiency tolerance in Tongzhou916 at 5d.

**Figure 2:**
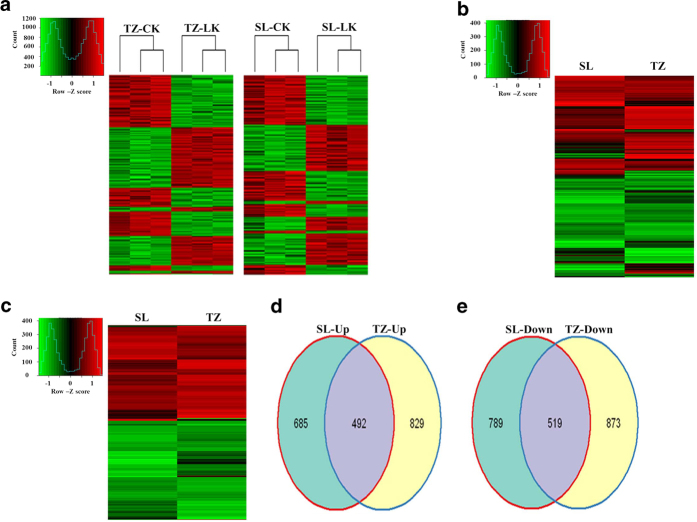
Hierarchical cluster and Venn diagrams of differentially expressed genes in wheat response to K^+^-deficient conditions. (**a**) Hierarchical cluster analysis of differentially expressed genes in Tongzhou916 (TZ) and Shiluan02-1(SL) during K^+^-sufficient (CK) and K^+^-deficient (LK) treatments. (**b**) Hierarchical cluster analysis of the differentially expressed genes (DEG) using averaged values of replicates in Tongzhou916 (TZ) and Shiluan02-1(SL) during K^+^ deficiency. (**c**) Hierarchical cluster analysis of the shared DEG using averaged values of replicates in TZ and SL during K^+^ deficiency. Venn diagrams of up-regulated (**d**) and down-regulated genes (**e**) under K^+^-deficient conditions.

Hierarchical cluster method and Venn diagram were performed to study the commonness and individuality of transcriptome features in Tongzhou916 and Shiluan02-1 under different potassium treatments (Fig. 2). There were more up-regulated genes in Tongzhou916 than that in Shiluan02-1 under K^+^-deficient conditions. There were a certain number of genes up-regulated only in Tongzhou916 (Fig. 2a,b). The numbers of up-regulated and down-regulated genes in Tongzhou916 were 144 and 84 more than those in Shiluan02-1 (Fig. 2d,e). This suggested that more genes might be stimulated under 5-d K^+^-deficiency in Tongzhou916. In addition, there were 492 up-regulated genes and 519 down-regulated genes exhibited transcriptome changes in both wheat genotypes, which might make contributions to the response to K^+^-deficiency among the all wheat genotypes. From the hierarchical cluster of the shared DEG between the two genotypes (Fig. 2c), 70% of up-regulated genes had the higher expression levels in Tongzhou916 than those in Shiluan02-1, while less than 50% of down-regulated genes had the lower expression levels in Tongzhou916 than those in Shiluan02-1. In other words, most shared up-regulated genes showed higher expression levels in Tongzhou916 and over half the shared down-regulated genes displayed lower down-regulation degree in Tongzhou916.

### Functional annotation of shared genes in two wheat genotypes response to K^+^ deficiency

**Figure 3:**
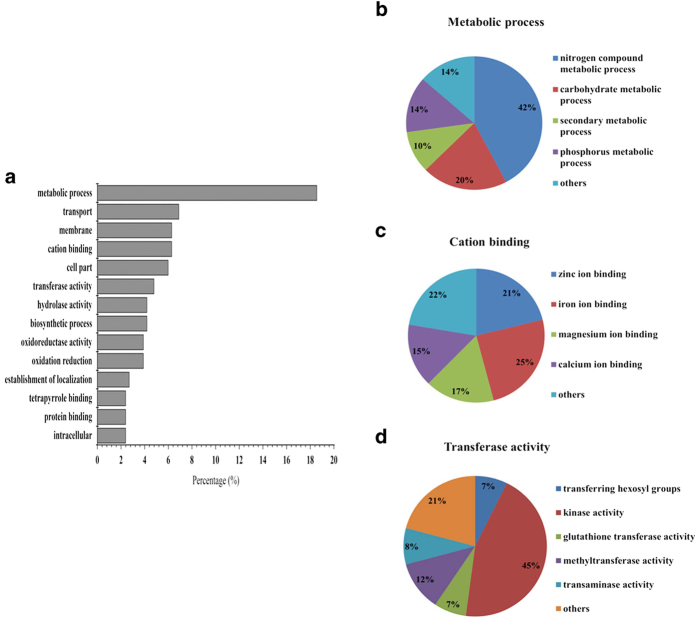
Functional category distribution of differentially expressed genes shared in the two wheat genotypes under K^+^-deficient conditions. (**a**) Percentages of differentially expressed genes in 14 main functional categories are shown. Detailed categories of differentially expressed genes in metabolic process (**b**) cation binding (**c**), transferase activity (**d**)

To classify differentially expressed genes of the two wheat genotypes according to the Gene Ontology (GO) terms, functional annotation was carried out. The 1011 shared genes, which showed significant changes under K^+^-deficient conditions in Tongzhou916 and Shiluan02-1 (*P* < 0.05), were divided into 14 main functional categories, according to the GO principles (Fig. 3a). Metabolic process (19%) was prominent among 14 main functional categories, followed by transport (7%), membrane (6%), cation binding (6%) and transferase activity (5%). Most of the above processes were thought to be associated with the response to K^+^-starvation closely. The GO analysis will provide the basis for us to better understand the transcriptome response to K^+^-deficiency in wheat roots.

Through further analysis of the metabolic process category, we found that about 42% genes in this category were related to nitrogen compound metabolic process, 20% to carbohydrate metabolic process, and 14% to phosphorus metabolic process (Fig. 3b). Many metabolic enzymes were involved in wheat response to K^+^-deficiency. Three probe sets encoding pyruvate decarboxylase isozyme were up-regulated under K^+^-deficient conditions (Table 2). Moreover, two probe sets (Ta.1870.1.S1_a_at and Ta.25990.1.A1_x_at) with putative functions in glutamate dehydrogenase were up-regulated, while one probe set (Ta.28435.1.S1_at) with putative functions in glutamate synthase was down-regulated under K^+^-deficiency. In addition, many other metabolic enzymes encoded genes, such as genes encoding sucrose synthase, ATPase and phosphatases were up-regulated. This suggested that genes encoding the regulation of metabolic enzymes might play a key role in wheat acclimation to K^+^-deficiency. For phosphorus metabolic process category, 24 genes encoding phosphorylation (11 up-regulated and 13 down-regulated) (Table 2), were transcriptionally regulated by K^+^-starvation.

In the category of cation binding, iron, zinc, magnesium and calcium binding proteins were included in the differentially expressed genes (Fig. 3c). There were 15 genes encoding peroxidase exhibited expressional changes in wheat response to K^+^-deficiency (including 8 down-regulated genes and 7 up-regulated genes), accounting for 71% of the iron-binding protein genes. We also identified 13 genes encoding calcium sensor protein, which showed transcriptional changes under K^+^-deficiency (5 up-regulated and 8 down-regulated) (Table 2).

In the transferase activity category, the main sub-category was kinase activity (45%) (Fig. 3d). Protein kinase as the main sub-category in kinase, is a kind of enzyme that catalyses protein phosphorylation. In this study, 31 genes encoding protein kinase were transcriptionally regulated (12 up-regulated and 19 down-regulated) under K^+^-deficiency (Table 2). The present result suggested that the process of phosphorylation and dephosphorylation might contribute a lot to the K^+^-deficiency management of wheat root. Methyltransferase is a kind of transferase, which can catalyze methyl into an acceptor molecule. In our microarray experiments, 11 genes encoding methyltransferase (5 up-regulated and 6 down-regulated) (Table 2), accounting for 12% of transferase activity category, showed changes in expression levels under K^+^-deficiency.

For ion transporters, three genes encoding nitrate transporter (Ta.5174.3.S1_x_at, Ta.8619.1.A1_at and Ta.21127.1.S1_at) were down-regulated after 5d of K^+^-starvation. In addition, the expression levels of two genes encoding peptide transporter (Ta.25793.1.S1_at and Ta.30027.1.S1_at) and one gene encoding ammonium transporter (Ta.27312.1.S1_x_at) also decreased under K^+^-deficient conditions (Table 2).

### Functional annotation of specific K^+^-responsive genes in two wheat genotypes

**Figure 4:**
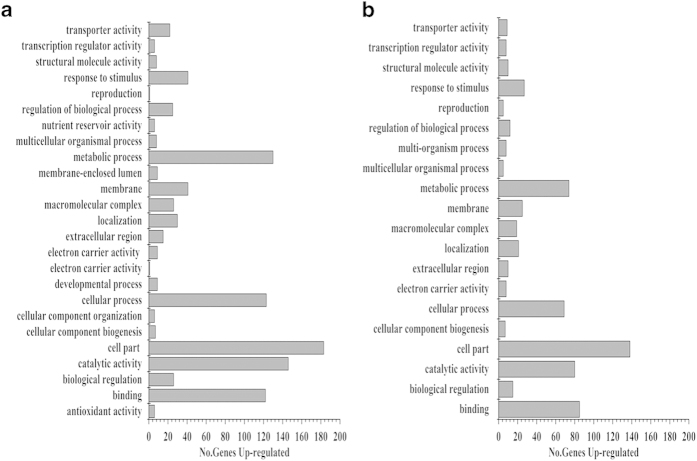
Numbers and Gene Ontology (GO) classifications of differentially expressed genes unique in the two wheat genotypes under K^+^-deficient conditions. (**a**) Functional category distribution of differentially expressed genes unique in Tongzhou916 (TZ). (**b**) Functional category distribution of differentially expressed genes unique in Shiluan02-1(SL).

In order to investigate the functions of specific K^+^-responsive genes in two wheat genotypes, GO analysis was carried out to compare the functional differences of specific up-regulated genes between two wheat genotypes. There were more specific up-regulated genes in Tongzhou916 (TZ) than those in Shiluan02-1 (SL) (Fig. 4), which suggested that more up-regulated genes had taken part in responding to K^+^-depletion in Tongzhou916. Although the main functions of two wheat genotypes were almost the same, the gene number of the main functions in Tongzhou916 was considerably higher than that in Shiluan02-1. Moreover, the function abundance of specific up-regulated gene in Tongzhou916 was higher than that in Shiluan02-1. There were five specific functions in Tongzhou916, including developmental process, cellular component organization, nutrient reservoir activity, antioxidant activity, and membrane-enclosed lumen, which were not found in Shiluan02-1.

For metabolic process category, there were three genes with function in jasmonic acid biosynthesis (Ta.8990.1.S1_at, Ta.1207.1.S1_x_at, and Ta.1207.1.S1_s_at) differentially expressed in Tongzhou916, while only one gene with function in jasmonic acid biosynthesis (Ta.20532.1.S1_at) in Shiluan02-1 (Table 3). In addition, there were three probe sets encoding vacuolar H^+^-pyrophosphatase up-regulated in Tongzhou916, while none probe set with such function showed changes in expression levels of Shiluan02-1. For catalytic activity, two probe sets encoding 1-aminocyclopropane-1-carboxylate oxidase (ethylene-related) were up regulated only in Tongzhou916. For response to stimulus category, we also found two jasmonic acid-related genes (Ta.8990.1.S1_at and Ta.27763.1.S1_at) in Tongzhou916, but none in Shiluan02-1. In addition, one ethylene-related probe set (Ta.8292.1.A1_at) and one probe set (Ta.25219.1.A1_at) encoding auxin response were found differentially expressed in Tongzhou916, while none was found differentially expressed in Shiluan02-1. Moreover, there were seven genes with functions in defense response (Ta.5720.1.S1_at, Ta.3828.2.S1_x_at, Ta.13907.2.S1_a_at, Ta.3590.1.S1_s_at, Ta.4328.1.S1_x_at, Ta.3467.2.S1_x_at and Ta.14281.1.S1_at) differentially expressed in Tongzhou916, while only one gene with functions in defense response (Ta.27229.1.S1_at) in Shiluan02-1 (Table 3). For transporter category, *TaHKT1* and *TaHAK2* genes were up-regulated in Tongzhou916, while only one high-affinity K^+^ transporter gene (*TaHKT1*) was up-regulated in Shiluan02-1.

For developmental process category, there were six genes with functions in anatomical structure development (Ta.6556.1.S1_x_at, Ta.1042.1.S1_x_at, Ta.6556.1.S1_at, Ta.1574.1.S1_s_at, Ta.449.1.S1_at, and Ta.19563.1.S1_at) differentially expressed in Tongzhou916, none in Shiluan02-1 under K^+^-deficiency (Table 3). For nutrient reservoir activity category, which played a role in the storage of nutritious substrates, six related genes (Ta.9402.1.S1_x_at, Ta.722.1.A1_at, Ta.87.1.S1_at, Ta.25181.1.S1_at, Ta.87.1.S1_x_at, and Ta.169.1.S1_x_at) differentially expressed in Tongzhou916, not in Shiluan02-1 under K^+^-deficiency. For antioxidant activity category, five peroxidase activity-related genes (Ta.8292.1.A1_at, Ta.22602.1.S1_a_at, Ta.21505.1.S1_at, Ta.22602.2.S1_x_at, and Ta.21137.1.S1_x_at) among the specific genes were only differentially expressed in Tongzhou916 under K^+^-deficiency (Table 3).

### Real-time fluorescence quantitative PCR (qRT-PCR) analysis

**Figure 5:**
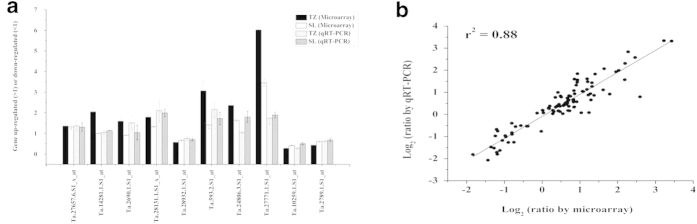
Verification of the transcriptome results through the experiments of qRT-PCR. (**a**) Ten random genes were selected from Tongzhou916 (TZ) and Shiluan02-1(SL). The vertical axis represents quantitative variations of the selected genes, while horizontal axis represents probe names of the selected genes. Data are means ± SE (n = 3). (**b**) The relationships between qRT-PCR and microarray of the shared genes, specific genes and randomly selected genes. Values are the log_2_ ratio (K^+^-deficiency/K^+^-sufficiency) for genes. The determine coefficient (r^2^) is indicated in the figure. All qRT-PCR reactions were performed in three biological replicates.

In order to verify the accuracy of microarray data, we chose ten genes randomly from the two wheat genotypes to perform qRT-PCR analysis. We designed specific-primers for the ten genes (see Supplementary Table S1). Quantitative variations of the selected genes between qRT-PCR and microarray were roughly similar (Fig. 5a), confirming that the results of microarray were reliable. In addition, we performed qRT-PCR analysis of the shared and specific genes mentioned in this paper. As shown in Fig. 5b, qRT-PCR results revealed that gene expression trends were significantly similar (r^2^ = 0.88) with those from the microarray data, indicating that our microarray results were reliable.

## Discussion

Valuable information about low K^+^-responsive genes has been shown above, which indicates that various physiological processes are involved commonly in wheat response to K^+^-deficiency and there are obvious transcriptional differences between the two wheat genotypes in response to K^+^-deficiency. Therefore, the Discussion section will focus on the following two questions: Which main physiological processes are involved commonly in wheat acclimation to K^+^-deficient conditions? Which special physiological processes or genes make Tongzhou916 more tolerant to low K^+^ stress?

Previous studies have emphasized the regulation role of some metabolic processes in plant adaptions to K^+^-deficiency^11^,^12^,^16^. The regulation of phosphorylation takes part in the modification of some transporters/channels for nutrient uptake. This mechanism can protect plants against the toxic damage caused by over accumulation, or provide energy for acquisition rate if needed^17^. Lee *et al.*^18^ reported that some proteins together with protein phosphatases made up the phosphorylation and dephosphorylation of protein, which played a key role in the K^+^ transport regulation. In the present data, several genes encoding phosphatase and phosphorylation were transcriptionally regulated, which indicated that phosphorylation and dephosphorylation also contributed a lot to wheat adaption to K^+^-deficient conditions. According to the previous study, the activity of OsAKT1 (as well as AtAKT1) is phosphorylated upon K deficiency^9^. In response to a signal event of K^+^-deficiency, phosphorylation may stimulates some K^+^ channels in wheat root, while this signal incident may be momentary and restrained by a signal terminator, which will lead to dephosphorylating, namely deactivation and recovery of K^+^ channels to the original state.

Metabolic enzymes contribute a lot to *Arabidopsis* acclimation to K^+^-deficient conditions^16^. Besides genes encoding phosphatase, several genes encoding other metabolic enzymes (such as ATPase, glutamate-related enzyme, sucrose synthase and pyruvate decarboxylase isozyme) had transcription regulation in wheat response to K^+^-starvation. ATPase activity is stimulated directly by external K^19^. Potassium channels together with the proton ATPase dominate the electric conductance of the plasma membrane and, therefore, their relative conductance determines the membrane potential, affecting the driving force for K^+^ movement in turn^20^. According to the present data, four genes encoding ATPase were up-regulated under K^+^-deficiency, which could lead to the increase of ATPase in wheat root. ATPase can improve the ATP hydrolysis and energy release, which can provide a driving force for transmembrane transport of potassium. Thus the up-regulations of genes encoding ATPase may improve K^+^-deficiency tolerance in wheat root. Glutamate may directly regulate ion channels of the putative glutamate receptor family^20^. Glutamate may affect cation transportation probably because that glutamate can cause large membrane depolarization of root cells and a change of cytosolic Ca^2+^ concentration^21^. In this study, two genes with putative functions in glutamate dehydrogenase were up-regulated, while one gene with putative functions in glutamate synthase was down-regulated under K^+^-deficiency. These glutamate-related enzyme genes can lead to the decrease of glutamate in wheat root, which result is similar to the study on K^+^-starved *Arabidopsis*^20^. Thus we can hypothesize that glutamate may involve in K^+^-deficiency signaling of wheat root cells under K^+^-deficient conditions. Many other metabolic enzymes showed marked changes when plants were in the face of K^+^-deficient conditions, these may be due to the K^+^-induced transcriptional variations in genes encoding metabolic enzymes.

A previous research indicated that peroxidases (heme-containing proteins) contributed a lot to *Arabidopsis* response to K^+^-deficient conditions^11^. One reason for this was likely that peroxidases were involved in cell wall responses associated with growth. Another reason for the participation of peroxidases in adaption to K^+^-starvation was likely to be their contributions to ROS detoxification, for H_2_O_2_ played a role of signal in K stress sensing^22^. Among the shared genes, 15 genes encoding peroxidase, accounting for more than two-thirds of the iron ion binding genes, showed changes in transcription under K^+^-deficiency. These genes may participate in growth-related cell wall responses and ROS detoxification of wheat root, and thus make contributions to wheat acclimatization to K^+^-starvation. Among the unique genes, several peroxidase-related genes were only found up-regulated in Tongzhou916, which may take a positive part in root growth and ROS production. Thus, the up-regulations of peroxidase-related genes may make a great contribution to promoting low-K^+^ tolerance of Tongzhou916. Previous studies found large proportion of Ca^2+^-binding proteins among K-regulated transcripts, which strongly suggested that intracellular Ca, to some extent, participated in responding to K^+^-deficiency^11^,^12^. In our microarray data, 13 calcium sensor protein genes were also found participating in wheat response to K^+^-deficient conditions, indicating that these genes might link calcium signaling and downstream target proteins in wheat adaption to K^+^-starvation.

Previous researchers found that many protein kinase genes differentially expressed under ionic stress^23^,^24^. Protein kinases regulated the transportation of plant K^+^, which was the fact revealed by some previous studies^25^,^26^,^27^. In the present data, a number of genes encoding protein kinase exhibited variations in expression levels of wheat under K^+^-deficient conditions. So we hypothesized that protein kinase encoding genes, which showed expression changes under K^+^-deficiency, might participated in wheat adaption to K^+^-starvation. These protein kinases were likely to perceive external low-K^+^ condition and regulate ion transport through phosphorylating ion transporters in plant cells, and thus enhanced transporter-mediated K uptake in wheat roots. Armengaud *et al.*^11^ found that the expression levels of some methyltransferase or methyltransferase-like proteins genes showed changes under K^+^-deficient and K^+^-resupplied conditions. Transfer of methyl groups via S-adenosyl-methyltransferase appears in some hormonal pathways (e.g. JA pathway). We also found several genes encoding methyltransferase showed changes in expression levels under K^+^-deficiency. These genes may affect the transfer of methyl groups and thus influence some K^+^-related hormonal pathways. So we can hypothesize that methyltransferase maybe another low K^+^-responsive transferase.

Previous researches on *Arabidopsis* and rice reported that several nitrate transporter genes were down-regulated during K^+^-deficiency^11^,^12^. Interestingly, several genes encoding peptide transporters also took part in the above response^11^. By contrast, the lack of N made the expression levels of potassium transporter genes increase^28^. Moreover, a similar relationship between NH_4_^+^ and K^+^ was found in barley and *Arabidopsis*^29^,^30^. In the present research, some genes encoding nitrate, peptide and ammonium transporters were down-regulated upon K^+^ starvation, which suggested some subtle relationship between K^+^ uptake and N transport. *AtNRT1.5* mRNA levels were down-regulated when potassium was limiting, suggesting that root-to-shoot nitrate transport is controlled by potassium levels^31^. Xia *et al.*^32^ found that OsNPF2.4 functions in acquisition and long-distance transport of NO_3_^−^, and that altering its expression has an indirect effect on K recycling between the root and shoot. These findings, suggesting that nitrogen metabolites were likely to take part in the signaling of low-K^+^ stress, provide a basis for molecular research on the relationship between potassium and nitrogen. Various high-affinity K^+^-transporter genes (such as *OsHAK1*, *HvHAK1*, *TaHKT1*, and *AtHAK5*), derived from different plants, are stimulated by K^+^-deficient treatment^6^,^7^,^8^,^33^. Moreover, from monocotyledons, OsAKT1^9^ and OsHAK5^10^ have been functionally characterized in plant to demonstrate their key roles in K acquisition from low K supplied culture medium. It may be a direct and effective strategy that K^+^ transporter genes will up-regulate in overcoming K^+^-deficient conditions. According to the present data, *TaHKT1* was up-regulated in both wheat genotypes. In addition, *TaHAK2* (a member of the KUP/HAK/KT family) was only up-regulated in Tongzhou916 under K^+^-starvation. Haro *et al.*^34^ reported that *PpHAK2* might encode a K^+^/H^+^ antiporter or a K^+^-H^+^ symporter, which regulated the H^+^ transportation between endoplasmic reticulum lumen and cytosol. Under K^+^-starvation, the increased expression level of *TaHAK2* may contribute to the strong low K^+^-tolerance of Tongzhou916.

Several jasmonic acid-related genes were found *Arabidopsis* root responding to K^+^-deficiency and K^+^-resupply^11^. Our data showed that jasmonic acid-related genes in Tongzhou916 were more abundant than those in Shiluan02-1. Jasmonic acid contributes a lot to the potassium signaling and management via various physiological and metabolic processes under low K^+^-stress. For example, jasmonic acid may make plants adapt to low-K^+^ stress through nutrient storage and remobilization^11^. Jasmonic acid may also reduce the pathogen attack in K-starved plants, which can also make decrease in damages from K^+^ starvation^20^. Therefore, more jasmonic acid-related genes in Tongzhou916 can make better low-K^+^ stress acclimation for Tongzhou916.

V-PPase can connect the H^+^-transmembrane transport and free energy generated by hydrolysis process of inorganic pyrophosphate (PPi), which makes V-PPase more predominant in the young and developing cells^35^. In additon, V-PPase can build the transmembrane electrochemical gradients, providing a driving force for the active transmembrane movement of various solutes (e.g. cation)^36^. Moreover, a previous study reported that V-PPase also took part in the transmembrane transportation of potassium^37^. In this study, shared genes encoding V-PPase were only up-regulated in Tongzhou916, which might improve the root K^+^ uptake in Tongzhou916. In addition, *Arabidopsis* V-PPase (*AVP1*) promotes the flow of auxin, which regulates the growth of root system^38^. The root of Tongzhou916 was better developed than that of Shiluan02-1 under low-K^+^ stress (Supplementary Table S2). Therefore, we can hypothesize that these genes encoding V-PPase may involve in the regulation of root development and root K^+^ uptake in Tongzhou916, and thus enhance the low-K^+^ tolerance of Tongzhou916.

Ethylene makes a great contribution to the development of root system^39^. Previous microarray data suggested that genes encoding ethylene showed changes in *Arabidopsis* response to low-K^+^ stress^22^. Ethylene also stimulated the development of primary root and root hair, which enhanced the *Arabidopsis* tolerance to K^+^-deficient conditions^40^. In the present data, shared genes encoding 1-aminocyclopropane-1-carboxylate oxidase, which took part in biosynthesis of ethylene, were only up-regulated in Tongzhou916. Ethylene responsive gene also showed changes only in Tongzhou916 after 5d K^+^-starvation. Auxin is important for root architecture^41^. Among the shared genes, IAA9-auxin-responsive gene, an IAA family member, only showed increase expression levels in Tongzhou916. For root architecture can be modified by the interactions between ethylene and auxin, ethylene-related and auxin-related genes may contribute a lot to the difference of root architecture between Tongzhou916 and Shiluan02-1 (Supplementary Table S2). Better root development made Tongzhou916 more tolerant to low-K^+^ stress.

For phosphorus and nitrogen starvation, several defense-related genes were transcriptionally regulated in *Arabidopsis*^42^,^43^. Some plant defensin genes were also found transcriptional changes in *Arabidopsis* under K^+^-starvation. The increase jasmonic acid in K^+^-starved plants will have certain effects on inducing defense response^11^. Defense response can prevent plants from insect and pathogen damages, which enemies K^+^-starved plants are likely to face^44^,^45^. Our microarray data showed that there were more genes encoding defense response remarkably up-regulated in Tongzhou916 under K^+^-deficient conditions, which maybe an important factor protecting Tongzhou916 under long-term K^+^-starvation.

Anatomical structure traits refer to the arrangement of the tissues and cells, and the characteristics of plant internal structure. Root anatomical structure, such as cortical size, the number and arrangement of root cells, determine the pathway and rate of nutrient uptake from the soil solutes to the root vascular cylinder^46^. Postma and Lynch^47^ reported that small changes in root anatomical structure were likely to affect physiology and growth of the whole plant, which had possible value for K^+^-uptake. From our microarray data, there were six genes encoding anatomical structure development showed transcriptionally changes in Tongzhou916, while none was in Shiluan02-1. These findings suggested that Tongzhou916 might adapt itself to K^+^-starvation through anatomical structure development.

Plants accumulate nutrients in the form of polysaccharides and proteins in appropriate organs and assimilate them into a wide variety of compounds for their growth and organogenesis. Nutrient reservoir (storage protein) is important for the storage of nutritious substrates. The existence of nutrient reservoir not only prevents the loss of nutrients, but also provides necessary nutrient for plants in the new growing season^48^. Armengaud *et al.*^11^ reported that several genes encoding vegetative storage protein took part in *Arabidopsis* response to external K^+^-levels. In this study, several genes encoding nutrient reservoir were up-regulated in Tongzhou916 after 5d K^+^-starvation, while none gene with such function was up-regulated in Shiluan02-1. This suggested that Tongzhou916 could better prevent nutrient loss via nutrient reservoirs during K^+^-starvation and thus made Tongzhou916 more tolerant to low-K^+^ stress.

In conclusion, these two wheat genotypes exhibited variant transcription features under K^+^-deficiency. There were more up-regulated genes involved in the response of low-K^+^ tolerant genotype to K^+^ starvation. The identified genes that responded to K^+^-deficiency belonged to metabolic process, cation binding, transferase activity, ion transporters and so forth. A lot of genes associated with jasmonic acid and defense response were considerably up-regulated, while the numbers of genes related to these processes in low-K^+^ tolerant genotype were higher than those in low-K^+^ susceptible genotype. In addition, some unique genes, such as vacuolar H^+^-pyrophosphatase, ethylene-related, auxin response, anatomical structure development and nutrient reservoir genes, were involved in low-K^+^ tolerant genotype. These unique genes played a key role in root architecture, K^+^ uptake and nutrient storage, which might contribute a lot to the strong low-K^+^ tolerance in wheat. However, further research is required to make it clear that how these specific genes regulate potassium metabolism.

## Methods

### Plant materials and low-K^+^ stress treatment

Two wheat genotypes, low-K^+^ tolerant “Tongzhou916” and low-K^+^ susceptible “Shiluan02-1” were used in this study. Two wheat genotypes were cultured hydroponically in an artificial climatic box. Environmental conditions of this box were as follows: 16/8 h day/night regime, 25/18 °C temperature, 70% atmospheric humidity and 30000 lux illumination intensity in the daytime. The normal nutrient solution was as follows (mmol L^−1^): Ca(NO_3_)_2_•4H_2_O 1.0, MgSO_4_•H_2_O 1.0, NaH_2_PO_4_ 0.25, NH_4_NO_3_ 1.0, CaCl_2_ 1.5, Fe-EDTA 0.1, MnSO_4_•H_2_O 1.0 × 10^−3^, ZnSO_4_•7H_2_O 1.0 × 10^−3^, CuSO_4_•5H_2_O 5.0 × 10^−4^, (NH_4_)_6_ Mo_7_O_24_•4H_2_O 5.0 × 10^−6^, H_3_BO_4_ 1.0 × 10^−3^ and K_2_SO_4_ 1.0, (pH 6.5). Nutrient solution was ventilated 12 h by pumps each day and replaced every day.

In the first experiment, K^+^-sensitivities of the two wheat genotypes were expected to be validated under low-K^+^ conditions. Two wheat genotypes were grown in normal and low-K^+^ conditions for two or three weeks, respectively. In this experiment, the concentration of K^+^ was 0.01 mM (K_2_SO_4_ 5.0 × 10^−3^ mmol L^−1^) in low-K^+^ conditions. Two wheat genotypes were harvested after two and three weeks. The growth states and K-sensitivity of the two wheat genotypes were then investigated. In the second experiment, we want to get the optimum time point of K^+^-deficient treatment for investigating the transcriptome differences in wheat roots during K^+^-starvation. In this experiment, wheat seedlings were firstly grown in normal nutrition solutions for 3 wk. Secondly, half of the above seedlings were transplanted to nutrient solution without K_2_SO_4_ (i.e. K^+^-deficient treatment (LK)), while another half were transplanted to nutrient solution with K_2_SO_4_ as control (i.e. control treatment (CK)). Wheat seedlings of the above two treatments were collected at 1, 3, 5 and 7 d for K-sensitivity assays. In the last experiment, the growth conditions were the same as that in the second experiment. Wheat roots of CK and LK were collected at 5d and quickly put into liquid nitrogen for following RNA isolation.

### Microarray analysis

The GeneChip experiments were finished with the assist of Shanghai Biotechnology Corporation. Total RNA was extracted using TRIZOL reagent (Cat#15596-018, Life technologies, Carlsbad, CA, US) following the manufacturer’s instructions and checked for a RIN number to inspect RNA integrity by an Agilent Bioanalyzer 2100 (Agilent technologies, Santa Clara, CA, US). Qualified total RNA was further purified by RNeasy micro kit (Cat#74004, QIAGEN, GmBH, Germany) and RNase-Free DNase Set (Cat#79254, QIAGEN, GmBH, Germany).

Total RNA were amplified, labeled and purified by using GeneChip 3’IVT Express Kit (Cat#901229, Affymetrix, Santa Clara, CA, US) followed the manufacturer’s instructions to obtain biotin labeled cRNA^49^. Array hybridization and wash was performed using GeneChip® Hybridization, Wash and Stain Kit (Cat#900720, Affymetrix, Santa Clara, CA, US) in Hybridization Oven 645 (Cat#00-0331-220 V, Affymetrix, Santa Clara, CA, US) and Fluidics Station 450 (Cat#00-0079, Affymetrix, Santa Clara, CA, US) followed the manufacturer’s instructions. Slides were scanned by GeneChip® Scanner 3000 (Cat#00-00212, Affymetrix, Santa Clara, CA, US) and Command Console Software 3.1 (Affymetrix, Santa Clara, CA, US) with default settings^50^.

### qRT-PCR Analysis

Part of the wheat roots, which were applied to perform gene chip detection, was applied for the qRT-PCR determination to verify the microarray results. TRIZOL reagent (Cat#15596-018, Life technologies, Carlsbad, CA, US) was used to extract total RNA of the root samples. The actin gene of *Triticum aestivum* L. was selected as the endogenous control. The extracted total RNA was applied to synthesize first-strand cDNA by cDNA synthesis Kit (Promega, USA) according to the manufacturer’s instructions. Three biological replicates (each biological replication contained twenty individuals) were performed in this experiment. qRT-PCR was performed using a 20 μl reaction system, containing 10 μl of 2X-RTmix, 2 μl template (0.2 μM), 1 μl forward primer, 1 μl reverse primer, and 6 μl nuclease-free water. PCR primers in this experiment (Supplementary Table S1) were designed by Primer 5 and DNAMAN software. Each PCR experiment was repeated at least for three times. The relative quantitative method of ΔΔ CT was applied to analyze the quantitative changes of the selected genes in the two treatments^51^.

### Biomass and K^+^ content measurement

The shoots and roots of different treatments were harvested at various times. The shoots and roots were collected separately, dried at 80 °C for 48 h, and then weighed (dry weight). K content in plant was determined by the method as described by Mills and Jones^52^, which involved digesting plant in a mixture of H_2_SO_4_ and H_2_O_2_. Potassium concentration in the digested solution was determined by a flame photometer. Three biological replicates were used for biomass and K^+^ content measurements. The K efficiency coefficient was calculated as follows^53^:

K efficiency coefficient = shoot dry weight under LK/shoot dry weight under CK (1)

### Statistical analysis

MAS 5.0 algorithm, Gene Spring Software 11.0 (Agilent technologies, Santa Clara, CA, US) was used to realize the normalization of data generated by the scanner. Only genes showing transcriptional differences (>1.5 fold-change and *P* < 0.05) were screened for further analysis. A positive or negative value represents up or down regulation, respectively. SBC Analysis System ( http://www.sas.ebioservice.com) and AgriGO online service ( http://bioinfo.cau.edu.cn/agriGO) were applied for the data analysis and functional annotation. T-test and ANOVA were carried out to test the significance of the type differences. For statistical analysis of data SPSS window version 17 (SPSS Inc., Chicago, USA) and Microsoft Excel (Microsoft Corporation, USA) were used. OriginPro 8.1 (Origin Inc., Chicago, USA) was used to draw the figures.

## Additional Information

**How to cite this article**: Ruan, L. *et al*. Comparative analysis of potassium deficiency-responsive transcriptomes in low potassium susceptible and tolerant wheat (*Triticum aestivum* L.). *Sci. Rep.* **5**, 10090; doi: 10.1038/srep10090 (2015).

## Supplementary Material

Supplementary Information

## Figures and Tables

**Table 1:** Numbers of differentially expressed genes (DEGs) in the two wheat genotypes under K ^+^ -deficient conditions.

**Genotypes**	**Gene Category**	**No. of Genes**	**Percentage of total DEGs**
Tongzhou916	Up	1321	48.69
	Down	1392	51.31
Shiluan02-1	Up	1177	47.36
	Down	1308	52.64

Up-regulated genes (Up) and down-regulated Genes (Down).

**Table 2:** Numbers of up- and down-regulated probe sets shared in the two wheat genotypes under K ^+^ -deficient conditions.

**Groups**	No. of LKR probe sets*	**Category**
Phosphorylation	11+13-	Metabolic process
Phosphatase	4+0-	Metabolic process
ATPase	4 + 0-	Metabolic process
Glutamate dehydrogenase	2 + 0-	Metabolic process
Glutamate synthase	0 + 1-	Metabolic process
Sucrose synthase	2 + 0-	Metabolic process
Pyruvate decarboxylase isozyme	3 + 0-	Metabolic process
Peroxidase	7 + 8-	Cation binding
Calcium sensor protein	5 + 8-	Cation binding
Protein kinase	12 + 19-	Transferase activity
Methyltransferase	5 + 6-	Transferase activity
Nitrate transporter	0 + 3-	Transporter
Peptide transporter	0 + 2-	Transporter
Ammonium transporter	0 + 1-	Transporter

^*^represents for total number of low K+-responsive (LKR) probe sets; “+” represents for up-regulated, “-” represents for down-regulated.

**Table 3:** Numbers of up-regulated probe sets unique in the two wheat genotypes under K^+^-deficient conditions.

**Groups**	No. of LKR probe sets*	**TZ**	**SL**	**Category**
Jasmonic acid-related	6	5	1	Metabolic process, Response to stimulus
Vacuolar H^+^-pyrophosphatase	3	3	0	Metabolic process
Ethylene-related	3	3	0	Catalytic activity, Response to stimulus
Auxin response	1	1	0	Response to stimulus
Defense response	8	7	1	Response to stimulus
Potassium transporter	3	2	1	Transporter
Anatomical structure development	6	6	0	Developmental process
Nutrient reservoir	6	6	0	Nutrient reservoir activity
Peroxidase-related	5	5	0	Antioxidant activity

^*^represents for total number of low K^+^-responsive (LKR) probe sets; “TZ” represents for “Tongzhou916”; “SL” represents for “Shiluan02-1”.
