# Supplementary material for: Comparative analysis of potassium deficiency-responsive transcriptomes in low potassium susceptible and tolerant wheat (Triticum aestivum L.)
Source: Sci Rep. 2015 May 18;5:10090. doi: 10.1038/srep10090 (PMC4650753; doi:10.1038/srep10090)

Comparative analysis of potassium deficiency-responsive transcriptomes in low potassium susceptible and tolerant wheat (*Triticum aestivum* L.)

Li Ruan[1], Jiabao Zhang[1*], Xiuli Xin[1], Congzhi Zhang[1], Donghao Ma[1] & Lin Chen[2]

[1] State Key Laboratory of Soil and Sustainable Agriculture, Institute of Soil Science, Chinese Academy of Sciences, Nanjing 210008, China
[2] Institute of Coastal Zone Research, Chinese Academy of Sciences, Yantai 264003, China.

[*]Corresponding Author
Prof. Jiabao Zhang
State Key Laboratory of Soil and Sustainable Agriculture
Institute of Soil Science
Chinese Academy of Sciences
Nanjing 210008, China
E-mail: jia-baozhang@hotmail.com
Tel: +8625 86881228
Fax: +8625 86881233

Supplementary Information

Supplementary Table S1: Primers used in qRT-PCR experiments.

Supplementary Table S2: Root morphology of the two wheat genotype under K^+ deficiency.

Supplementary Figure S3: Correlation coefficient (A) and cluster analysis (B) of 12 microarrays.

Supplementary Figure S4: The relationships between qRT-PCR and microarray of the shared genes and specific genes in Tongzhou916 (A) and Shiluan02-1(B).

Supplementary Table S1: Primers used in qRT-PCR experiments.

Probe ID	Forward primer (5'-3')	Reverse primer (5'-3')
Ta.27657.6.S1_x_at	AAGGTCGCCGCCAAGGAGTA	CATGAGAAATGGATCTAAGCAACAC
Ta.14281.1.S1_at	CGTCCCTACTCCGTCTATTTCCA	AATGCCGAGGGTGCAGGTAG
Ta.2690.1.S1_at	GAGCATCTCCCAGCAACCAT	GCAGGACAGGACACCATCAAC
Ta.28131.1.S1_x_at	TCTAAGATGAACCTATTTGGCGTA	AGCGAGGTAGATCAATGGTGG
Ta.28932.1.S1_at	GCAACAGATAACCTGATCCAGAAG	GTGCTCCACAGCCATGCCAT
Ta.593.3.S1_a_at	CCAAGCCCGACTATGCGACA	AAGAAGGTCCCGACAATAAGAGG
Ta.24806.3.S1_at	ATGGTTGCTGCTCATCTACTGC	CACTGGCTAGGAGCATAGGAAG
Ta.27771.1.S1_at	CCATCTATCTGGCTCAGGGTATC	CTTTGCGACCCCTCTCTGTAG
Ta.10259.1.S1_at	TCACCTGGCTCGACAACAAC	ACCCTGGTACATCTTGGCGA
Ta.2789.1.S1_at	GACCAAGGAAGGCCAAGACA	ATTGATTCTACGCCCAACAAAG
Ta.1042.1.S1_x_at	CCTGACACCAAAGTAACCGTAG	ATTCACCTCGTCTCAGCTCTATC
Ta.1207.1.S1_s_at	CTTGTTGCTTACGGGAGGAT	TGTGTAGCCAATGACAGGAT
Ta.87.1.S1_at	GAAGCCTACATGGTTGTCTCCT	CTATCCTTAGCGCCTTGGTG
Ta.21137.1.S1_x_at	GCGTCGGCACAGTTCAACAG	CAGCCTTATTATGTCGCCTTTG
Ta.13907.2.S1_a_at	GTGCTCGCCAAGAAGTACAGG	CGTAGCGGTCGGTGTTGTAG
Ta.1574.1.S1_s_at	GGTGCTCACCAAAATGGAAG	CTTCACGGTCACCTTCTGC
Ta.169.1.S1_x_at	TCTGGGAGAACAACCACTACTAAG	TTCACTGATTACAGACACACAATGG
Ta.1870.1.S1_a_at	CATCAAGGCCATGTGCAAGTCTC	GAGCAACCCGGTTCACTCCCAAG
Ta.19563.1.S1_at	CGTAAGCGAGCTATTAGAGTTTG	CGTATCCTTAACCCACCCATC
Ta.20532.1.S1_at	GGTTTGAGGTAGGCGCAGAG	ATACAGCCAGCGGTAAGAAAG
Ta.21127.1.S1_at	GCCACTGATCGATGCATGCTACTTG	ACATACATTATACGCCACCGAATAC
Ta.1207.1.S1_x_at	CGCAGGCAGATACCTCACG	CATAGCCATCGGCCACCAC
Ta.21505.1.S1_at	CCGACAACACGGTCAACAAC	CCCCTGAGAGCCAGTCAATG
Ta.22602.1.S1_a_at	CTTCTTGTTGGTCTTATGGCAG	GCTTCGAGCCTCCTTGATG
Ta.6556.1.S1_x_at	GGCTCCAACTCATCCGATTAG	GGCTCCAACTCATCCGATTAG
Ta.25181.1.S1_at	ATCCAACCCAAATAGGCTCTTC	TGGCTGCTGAGTGACGACAAC
Ta.25219.1.A1_at	AAGGGCGAGGGCGAGATGGT	AGCAGTGCCGTTCTGTTTG
Ta.25629.1.S1_at	GCCAGTTCCGTGACTCCTTG	GGGAATGCTCTGTCCGTTG
Ta.25793.1.S1_at	CCAGTCTGTAATGTAGGGTGTCGT	CTTCTTCAGCCAATCAACCAGTC
Ta.25990.1.A1_x_at	ACATCGACACCGTCATGGCCCA	CACAAACTTCTTCCACGCCTCC
Ta.27229.1.S1_at	GTCCGTCAGTAGTTTCTTCGTG	ACCGAAACGCCGACCGATTG
Ta.27312.1.S1_x_at	CATGCGTGCGTTTAGAGGA	ACCTTGAGCAGCCACCTATC
Ta.27763.1.S1_at	GCCAAGAACCCGCACTTTC	GTGAAGCCAGAGGAGCAGAG
Ta.28435.1.S1_at	GCTGCAATTTTGTGGAAGCTCTG	TTACAGGGGTGGTTGGTTACCT
Ta.30027.1.S1_at	GGCGATGAGGAGGTAGACG	CGTTGTTGCCAAAGTCACC
Ta.3467.2.S1_x_at	ACCCCGTCGTCTTCCTCCA	AACAGCACGATCCTGTAGAACTC
Ta.3590.1.S1_s_at	GGATCTGCCCCTTCTTGGAG	GTGGTTGGAGAAGGACACCTTG
Ta.3828.2.S1_x_at	GGCGTACCCTATCCAGTTCC	CCACGTTACACTTCACTCCT
Ta.4328.1.S1_x_at	ACCGCAACGTCGCACATCAA	TCCACGCCTGGCAGCATCTT
Ta.449.1.S1_at	CAGGGACTGGGACATCGACTAC	TCTGGAGCGGGATGGTGAAG
Ta.5174.3.S1_x_at	GGACGCCGACTACAAGAACGTGAAG	TGTCCTTCTTGAGGTCGTCGTGGGT
Ta.5720.1.S1_at	TGTGTTGCCTCTTCATCTGC	GGTCTCATAAGCCAACACTAAC

Ta.6556.1.S1_at	CAACTCATCCGATTAGGCTTG	CCTTGTAGGATTCTTCGCTCA
Ta.22602.2.S1_x_at	AAGTTGGGAGGATCTGACAAGTG	GTTGAATGCTGTTTCGCTTATG
Ta.722.1.A1_at	GACGACTCTGCTTGCTGTTG	ATATTGCACCCTCTGTTTCAT
Ta.8292.1.A1_at	CCCTTCGGACCAGGTGATG	CGGTCAGCGGGCTTATGTT
Ta.8619.1.A1_at	GTGGATCGCCGACGACCTCAACAAG	GGCGAAGAGGTAGACGACGAGGTTG
Ta.87.1.S1_x_at	GTCTCCTTCAACAGCCAGAAC	CACCAGCGAACTTGGACTTG
Ta.8990.1.S1_at	CTCATGGCCGTCCACATCTC	CGAAAGGAAACTAAGGAAAG
Ta.9402.1.S1_x_at	CCCAGGAGTGTAGCGTCTTC	ATTTCCGCATGGAGGATTGTG

Supplementary Table S2: Root morphology of the two wheat genotype under K^+ deficiency.

K levels	Genotypes	Total root length (cm)	Total root surface area (cm^2)	Total root volume (cm^3)	Average root diameter (mm)	Root tips
2 Mm	TZ	454.78±31.98**	47.90±3.57**	0.42±0.05**	0.34±0.01*	474.33±3.51**
	SL	209.48±33.78	19.86±4.89	0.15±0.02	0.29±0.02	236.33±20.03
0 Mm	TZ	390.73±8.02**	41.21±1.73**	0.35±0.03**	0.36±0.01**	433.67±5.86**
	SL	128.05±2.49	11.42±0.93	0.05±0.02	0.26±0.01	142.00±3.00

Results are means ±SD, n=3. * and ** denote significant differences at $P < 0.05$ and 0.01, respectively.

Supplementary Figure S3: Correlation coefficient (A) and cluster analysis (B) of 12 microarrays.

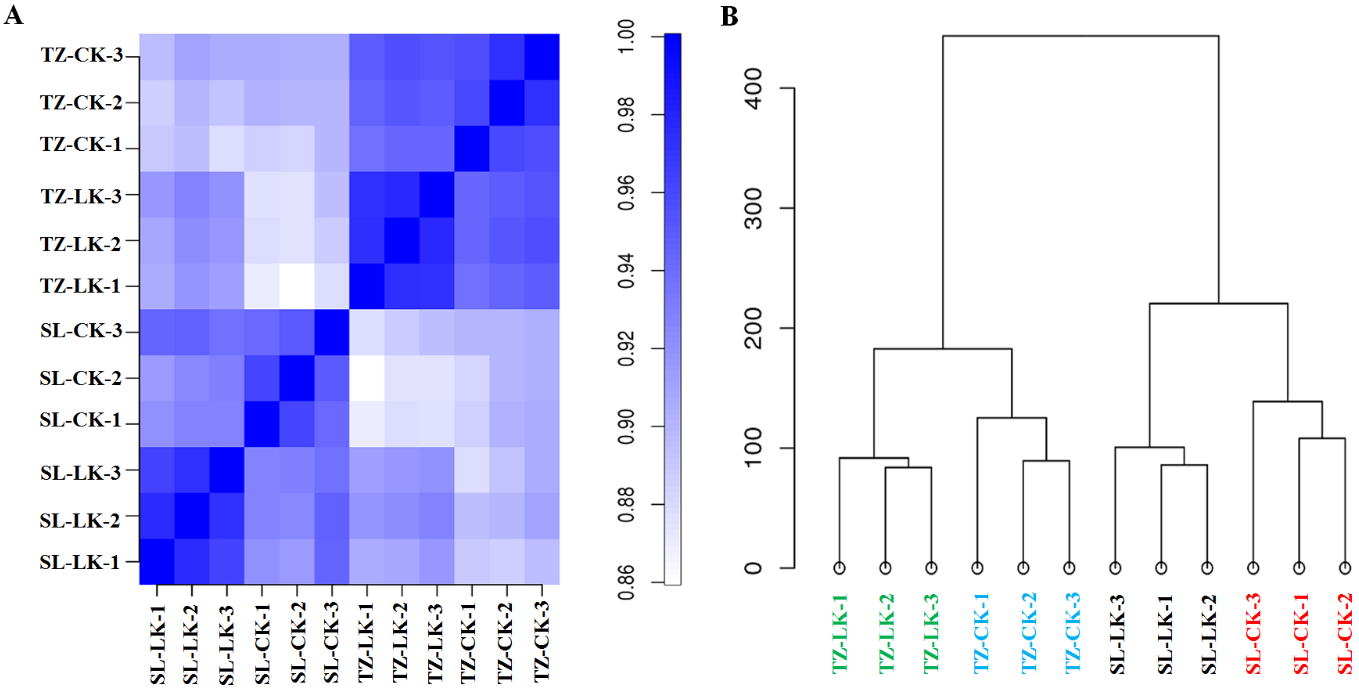

Supplementary Figure S4: The relationships between qRT-PCR and microarray of the shared genes and specific genes in Tongzhou916 (A) and Shiluan02-1(B).

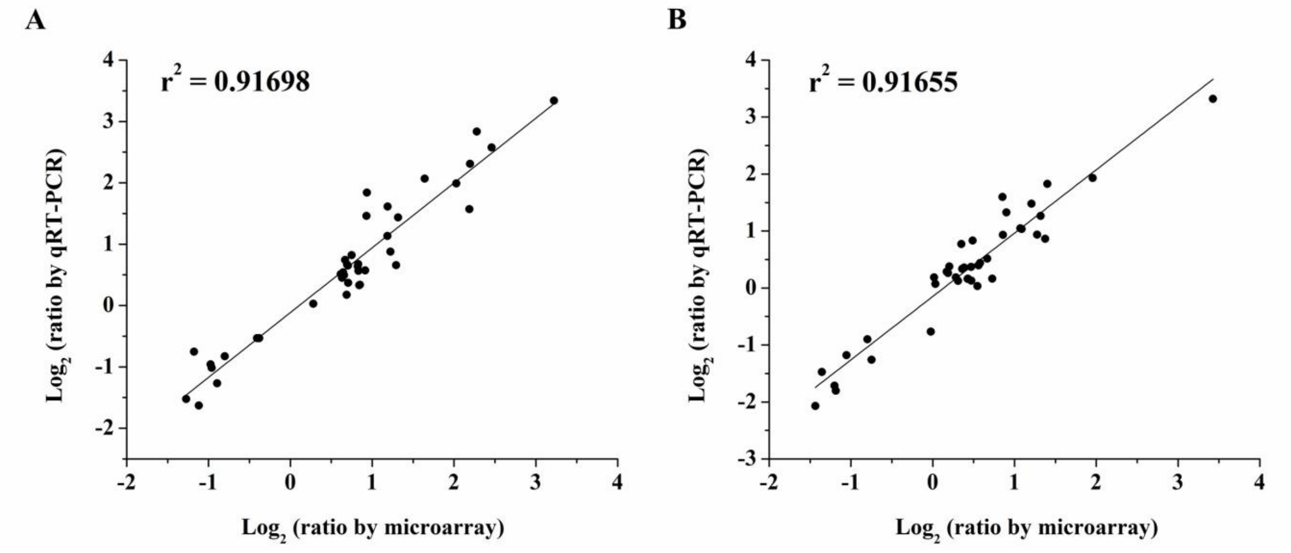

Supplement: Supplementary Information [file srep10090-s1.pdf]
